# Natural variation of photosynthetic efficiency in *Arabidopsis thaliana* accessions under low temperature conditions

**DOI:** 10.1111/pce.13811

**Published:** 2020-06-28

**Authors:** Aina E. Prinzenberg, Lucia Campos‐Dominguez, Willem Kruijer, Jeremy Harbinson, Mark G. M. Aarts

**Affiliations:** ^1^ Horticulture and Product Physiology Wageningen University and Research Droevendaalsesteeg 1 Wageningen 6708 PB The Netherlands; ^2^ Laboratory of Genetics Wageningen University and Research Droevendaalsesteeg 1 Wageningen 6708 PB The Netherlands; ^3^ Plant Breeding Wageningen University and Research PO Box 386 Wageningen 6700 AJ The Netherlands; ^4^ Royal Botanic Garden Edinburgh 20A Inverleith Row Edinburgh EH3 5LR United Kingdom; ^5^ Biometris Wageningen University and Research Droevendaalsesteeg 1 Wageningen 6708 PB The Netherlands; ^6^ Laboratory of Biophysics Wageningen University and Research Wageningen The Netherlands

**Keywords:** Arabidopsis, cold, GWAS, natural variation, photosynthesis

## Abstract

Low, but non‐freezing, temperatures have negative effects on plant growth and development. Despite some molecular signalling pathways being known, the mechanisms causing different responses among genotypes are still poorly understood. Photosynthesis is one of the processes that are affected by low temperatures. Using an automated phenotyping platform for chlorophyll fluorescence imaging the steady state quantum yield of photosystem II (PSII) electron transport (Φ_PSII_) was measured and used to quantify the effect of moderately low temperature on a population of *Arabidopsis thaliana* natural accessions. Observations were made over the course of several weeks in standard and low temperature conditions and a strong decrease in Φ_PSII_ upon the cold treatment was found. A genome wide association study identified several quantitative trait loci (QTLs) that are associated with changes in Φ_PSII_ in low temperature. One candidate for a cold specific QTL was validated with a mutant analysis to be one of the genes that is likely involved in the PSII response to the cold treatment. The gene encodes the PSII associated protein PSB27 which has already been implicated in the adaptation to fluctuating light.

## Abstract 2

We performed a quantitative genetics analysis on an *A. thaliana* diversity panel to identify genetic factors involved in photosynthetic cold response. Many QTLs were identified and we present the *PSB27* gene, encoding a protein associated with photosystem II repair, as a candidate for one of the QTLs.

## 1 INTRODUCTION

Low, but non‐freezing, temperatures (between 5 and 15°C) negatively affect the growth and development of most plants (Mckersie & Leshem, 1994). Plants growing in moderate climates are often exposed to low temperatures at some stage of their life and have evolved strategies to adapt to such unfavourable temperature conditions. These strategies can comprise changes in enzyme activity, osmolyte accumulation, membrane fluidity (Upchurch, 2008; Zheng, Tian, Zhang, Tao, & Li, 2011) or more specific changes to photosynthesis and energy metabolism (Hüner et al., 2012). The effect of low temperature on plants depends not only on the temperature, but also on other environmental factors to which the plant is simultaneously exposed (Crosatti, de Laureto, Bassi, & Cattivelli, 1999; Huner, Öquist, & Sarhan, 1998; Waraich, Ahmad, Halim, & Aziz, 2012), especially light intensity (Franklin, Toledo‐Ortiz, Pyott, & Halliday, 2014; Wanner & Junttila, 1999) and the developmental stage at cold exposure (da Cruz et al., 2013; Hatfield & Prueger, 2015; Nykiforuk & Johnson‐Flanagan, 1997). Considering the complexity of environmental cues and the differences in sensitivity to cold, it is not surprising that there is ample natural genetic variation for the response to cold, both between (Atkin, Loveys, Atkinson, & Pons, 2006) and within species (Barah et al., 2013; Bravo et al., 2007; Brüggemann, van der Kooij, & van Hasselt, 1992; Sanghera, Wani, Hussain, & Singh, 2011). Although many molecular components of the cold signalling and adaptation pathway have been identified (Cheng et al., 2007; Chinnusamy, Zhu, & Zhu, 2007), the genetic differences that give rise to the natural variation in cold responses between species or varieties are largely unclear. For example, one common pathway for low temperature perception and induction of freezing tolerance is the “C‐repeat/DRE‐Binding Factor” (*CBF*)—“Inducer of *CBF* expression 1” (ICE1) regulon. This pathway has been well described for *Arabidopsis thaliana* (see review: Miura & Furumoto, 2013) and seems to be conserved in other species (Choi, Rodriguez, & Close, 2002; Jaglo et al., 2001). The cold‐activated transcription factor ICE1 triggers the downstream transcription factor CBF3. The ICE1 and CBF transcription factors subsequently induce expression of cold responsive (*COR*) genes. These *COR* genes code for a variety of proteins needed for cold adaptation, for example those that cause osmotic changes, changes in membrane stability, or that act as chaperones (Plieth, Hansen, Knight, & Knight, 1999; Sangwan, Foulds, Singh, & Dhindsa, 2001; Usadel et al., 2008). However, the variation in *CBF*‐ and *COR*‐genes identified so far cannot explain all the variation seen between different accessions in *A. thaliana*, at least in terms of their freezing tolerance (Gery et al., 2011; McKhann et al., 2008); similar studies in cold but non‐freezing conditions are missing.

One possible option for determining the genetic regulation underlying variation in low temperature adaptation is to study the natural genetic variation in cold response between wild accessions of a species by genetic linkage or association mapping. Natural variation for freezing tolerance has already been studied for *A. thaliana* (Hannah et al., 2006; Mishra, Heyer, & Mishra, 2014) but not for cold, non‐freezing temperatures. *A. thaliana* grows in diverse habitats and shows heritable differences stress responses among the different accessions (Alonso‐Blanco et al., 2005; Lefebvre, Kiani, & Durand‐Tardif, 2009). A HapMap population consisting of a diversity panel with ~360 accessions, representing the species‐wide natural genetic variation, has been developed for Genome Wide Association Studies (GWAS) in *A. thaliana* (Li, Huang, Bergelson, Nordborg, & Borevitz, 2010). This population offers several interesting features for genetic analysis: it is based upon a publicly available set of accessions that have been genetically characterized and selected for diversity and minimal relatedness (Baxter et al., 2010; Horton et al., 2012). In addition, well‐developed statistical tools for quantitative genetic analysis (Kruijer et al., 2015; Seren et al., 2012) as well as extensive genome information are available (TAIR www.arabidopsis.org; Araport www.araport.org) for *A. thaliana*. A SNP array with approximately 250,000 SNP makers (http://1001genomes.org/; Kim et al., 2007) at an average density of about one SNP per 500 bp (Atwell et al., 2010) provides a high genome coverage that enables the selection of a small number of candidate genes for loci identified in a GWAS. Since *A. thaliana* is predominantly self‐fertilizing and lines have been propagated for several generations after collection, the accessions are reasonably assumed to be homozygous which means that replicate experiments with the same genotypes can be performed (Koornneef, Alonso‐Blanco, & Vreugdenhil, 2004; Weigel & Mott, 2009). This enables the comparison of genotypic performance in different environmental conditions. Genes involved in the response to environmental changes have already been identified by using the *A. thaliana* HapMap population in GWAS. For example, Baxter et al. (2010) found strong evidence that variants of the *HKT1* gene are involved in differences of leaf sodium accumulation, salt tolerance (Rus et al., 2006) and habitat distribution, while other studies provided candidate genes for the response to combined drought and herbivore stress (Davila Olivas et al., 2017) and a change in irradiance level (van Rooijen et al., 2017). The ability to compare the response of the HapMap population to different conditions has also allowed a multi‐trait mapping for 11 single and combined stresses that revealed common underlying genetics (Thoen et al., 2017).

Considering the high genetic resolution of the *A. thaliana* HapMap population, the success of quantitative genetics studies depends mostly on the quality of the phenotypic data. The trait measured needs to be representative of the stress effect and the precision of its quantification needs to be high. In response to low, non‐freezing temperature, differences in leaf biomass related traits, such as leaf size and other leaf morphological traits have been observed in *A. thaliana* (Armstrong, Logan, & Atkin, 2006; Gorsuch, Pandey, & Atkin, 2010). However, while these traits could be used for the quantification of the effect of cold, to allow the identification of differences between genotypes, their measurements need to be very precise, demanding high numbers of replicates, as the effect size is relatively small. This phenotyping is normally very labour intensive for large plant populations. In addition, measuring biomass‐related traits is often destructive and therefore can only be assessed at one time point. A solution to this would be the use of image‐based traits, for example the quantum yield of the photosystem II (PSII) electron transport under dark‐adapted conditions (maximum quantum yield, *F*_*v*_/*F*_*m*_) or under steady‐state illumination (the operating quantum yield of PSII, Φ_PSII_). These are integrative, primary physiological traits that have been found to be highly responsive to different stresses (Baker & Rosenqvist, 2004; Maxwell & Johnson, 2000), including low temperature exposure (Gray, Hope, Qin, Taylor, & Whitehead, 2003; Hurry, Krol, Oquist, & Huner, 1992). *F*_*v*_/*F*_*m*_ and Φ_PSII_ can be measured by chlorophyll fluorescence (CF) imaging, a fast technique that allows high‐throughput phenotyping (Harbinson, Prinzenberg, Kruijer, & Aarts, 2012; Rungrat et al., 2016; van Bezouw, Keurentjes, Harbinson, & Aarts, 2019). It is non‐invasive, allowing repeated measurements of the same plants, thus providing highly informative time‐course data. In a small rosette plant as *A. thaliana*, CF‐imaging of the projected leaf area allows the measurement of nearly the whole rosette surface. The technique enables robust quantification of the relative quantum yield for electron transport by PSII (Φ_PSII_), for which ample genetic variation has been found in *A. thaliana* (Flood et al., 2016; Mishra et al., 2014; van Rooijen, Aarts, & Harbinson, 2015). Therefore, Φ_PSII_ is an ideal trait to screen large numbers of in *A. thaliana* for the quantitative genetic analysis of low temperature responses.

In this study the *A. thaliana* HapMap population has been employed in a GWAS to identify loci involved in natural variation of the response of photosynthesis to low temperature. Using an automated high‐throughput phenotyping platform, the Phenovator (Flood et al., 2016), the response of all genotypes to a prolonged temperature decrease has been followed over time. Several loci specific to the low temperature condition that influence Φ_PSII_ are identified, and mutants were used to confirm the relevance of candidate genes residing at these loci.

## 2 MATERIAL AND METHODS

### 2.1 Plant material

Three or four replicates per genotype were evaluated for 347 lines of the *A*. *thaliana* HapMap population (Baxter et al., 2010) listed under N76309 at the Nottingham Arabidopsis Stock Centre, NASC; www.arabidopsis.info (Table S3). The T‐DNA insertion mutant lines (Table S2) were also ordered from NASC and when needed tested for the homozygosity of the T‐DNA insertion (full list of tested lines and primers in Table S2). Many of the tested T‐DNA insertion lines have a Col‐0 background, and in principle should be identical to N60000 (Col‐0), apart from the mutated locus. In practice, however, propagation over several years in different laboratories can lead to accumulation of other mutations (Ossowski et al., 2010) or introgression of other parental alleles due to accidental cross pollination. To be able to evaluate the effect of different genetic backgrounds of Col‐0 on Φ_PSII_, three Col‐0 lines (N60000, N907 and N76113) were characterized. No significant difference was detected in Φ_PSII_ between these lines in the cold or control temperatures (Figure S3). Therefore, all mutants with a Col‐0 background were compared to N60000. Only the mutants N851044 and N1001708 have the wild‐type background Col‐2 (N28170) and Col‐4 (N933), respectively, and have been compared to these accessions. The mutant N866141 is in a Col‐3 background but no phenotypic data on Col‐3 could be obtained and in this study it is compared to Col‐0. In the mutant analysis 12–24 replicate plants were evaluated per genotype, as not all mutants were characterized together but in two independent experiments (since only three replicates could be evaluated for N653579 in the initial experiment, the data from the quantitative complementation test with 103 replicates was used for Figure 3). For the complementation test the mutant line N653579 and Col‐0 (N60000) were both crossed (as mother plants) to the accessions Catania (Ct‐1; CS76114) and Siegen (Si‐0; CS28739). The resulting F1 seeds were used for the complementation test. Phenotypic outliers were removed: as plants from one N653579 × Ct‐1‐cross and N653579 × Si‐0‐cross were similar to the mutant and plants from one Col‐0 × Ct‐1‐cross were similar to Col‐0, it is assumed that the crosses failed. For Ct‐1 and Si‐0, 13 and 19 replicates were evaluated respectively, 51 replicates for Col‐0, 103 replicates for N653579, and 25–67 replicates were evaluated for the different F1plants.

### 2.2 Growing conditions and photosynthetic phenotyping

The *A. thaliana* seeds were placed on filter paper in a sealed petri dish and moistened with purified water. These dishes were kept at ~5°C in the dark for stratification for 3–5 days. Afterwards they were sown on 4 cm × 4 cm × 4 cm rock‐wool blocks (Grodan, Roermond, The Netherlands). For the Φ_PSII_ phenotyping experiment, the rock‐wool blocks were placed in a grid system (Flood et al., 2016) and flooded three times per week for 5 min with nutrient solution for irrigation. The nutrient solution is composed of macronutrients, obtained from Yara Benelux B.V. fertilizers (Rotterdam‐Vlaardingen, The Netherlands) and micronutrients from the Agrispoor product line of Horticoop B.V. (Bleiswijk, The Netherlands) to reach the following final elemental composition of dissolved ions: NH_4_ 1.7 mM, K 4.13 mM, Ca 1.97 mM, Mg 1.24 mM, NO_3_ 4.14 mM, SO_4_ 3.14 mM, P 1.29 mM, Fe 21 μM (composed half‐half of Fe‐DTPA and Fe‐EDDHSA), Mn 3.4 μM, Zn 4.7 μM, B 14 μM, Cu 6.9 μM, Mo 0.5 μM. The solution was adjusted with KOH or H_2_SO_4_ to a pH of 5.5, the final EC was 1.4. During the phenotyping experiment the plants were grown in a climate‐controlled growth room that was set at a day length period of 12 hr (from 8 to 20 hr), a light intensity of 200 μmol*m^−2^*s^−1^, night and day temperatures of 21°C and a relative air humidity of ~70%. On day 14 after sowing (14 DAS) the air temperature was reduced, over the course of 2 hours after light onset, to approximately 5°C and this low temperature (cold) was kept day and night. The plants were grown for 7 days in the cold and on day 21 after sowing (21 DAS), the air temperature was raised again to 21°C. The light was supplied by light tubes and bulbs (“Master TL5 HO” and “Superlux Agro” from Philips, Eindhoven, The Netherlands). The light tubes were increasing and decreasing in intensity over the course of half an hour at the beginning and end of each light period. For seed propagation and crosses the plants were grown on rock‐wool blocks in a greenhouse with supplemental lighting (at a 16 hr light period). Those plants were irrigated with a diluted nutrient solution (relative composition see above) or tap water.

### 2.3 Phenotyping

Over the course of the experiment three CF‐images were evaluated per day with an automated camera system that was moving over the plants (Flood et al., 2016). To measure all plants took 47 min and the three measurements were started at 12 hr, 16.30/15.30 (for the mapping population/for the mutant experiments; termed “17 hr”), and at 18.30 (termed “19 hr”). CF images were evaluated for a period from 2 days before up to 2 days after the cold treatment (12 DAS–22 DAS).

### 2.4 Statistical analysis

As in van Rooijen et al. (2017), the GWAS analysis and heritability calculations were performed with a mixed model in R (Kruijer et al., 2015) using 199,589 SNPs with a minor allele frequency threshold of at least 0.05. The Bonferroni threshold for the analysis is 6.6012 (calculated as: −log10 [minimum allele frequency/number of tested genetic markers]). As phenotypic value, the average Φ_PSII_ for each accession was used and the GWAS analysis was performed for each individual time point covering the measurement time between 12 DAS and 22 DAS. SNPs with an association of −log_10_(p)‐value ≥ 3 were plotted in a heat map (custom R‐script using the plot function in R version 3.3.2). Enlargements of the quantitative trait loci (QTLs) in Figure 2 were done with Preview (version 10.0, Apple Inc.) and EazyDraw (version 8.7.0, Dekorra Optics, LLC, Poynette, WI). Genetic regions were counted as candidate regions (named QTL 1 to 105, 90 or 94 in control, cold or recovery, respectively) if SNPs were significantly associated with the trait on at least three of the six time points in standard temperature (control and recovery, respectively) or at least 11 of the 21 time points in low temperatures. For selecting the candidate genes, a 30‐kb window on each side of the associated SNPs was chosen; in case those regions overlapped they were counted as one QTL. The broad sense heritability (H^2^) was calculated with the heritability package in R (Kruijer et al., 2015).

The difference between treatments was assessed with a Student's *t* test per genotype between control and low temperature conditions; differences between genotypes were tested per time point. For the assessment of how many genotypes of the HapMap had a reduced Φ_PSII_ upon cold treatment and how many recovered on 21DAS12h, plants were considered identical at a *p* > 0.05. Averages, standard deviation or error of the phenotypic values and *t*‐tests were calculated with Microsoft Excel. When average values of Φ_PSII_ were formed over several (N_i_) time points, the standard deviation was calculated from the individual standard deviations per time point (dx_1_, dx_2_, dx_i_) as sqrt((dx_1_^2^ + dx_2_^2^ + dx_i_^2^)/(N_i_ − 1)). The percentile change in Φ_PSII_ between control (C) and low temperature conditions (T) or between mutant (T) and wild type (C) were calculated as: ([C − T]/C)*100.

To test for the difference between the two pairs of F1 genotypes in the complementation test, N653579 × Si (“MS”) versus N653579 × Ct (“MC”) and Col × Si (“CS”) versus Col × Ct (“CC”), a contrast was defined as (mean(CC) – mean(CS) – mean(MC) + mean(MS)). The standard error was calculated from the mean sum of squares (MSSq) of the residuals from a linear model explaining the Φ_PSII_ by the genotype, following the formula: sqrt(MSSq * [1/N_CC_ + 1/N_CS_ + 1/ N_MC_ + 1/N_MS_]) in which N is the number of replicates per genotype group. The ratio of the contrast to the standard error was compared to the t‐distribution and a *p*‐value determined (two sided test). For Figure 4a a contrast with the absolute values is plotted: |mean(CC) – mean(CS)| – |mean(MC) – mean(MS)|. Figures 3 and 4 were obtained with the package ggplot2 (version 3.0.0; Wickham, 2016) in R (version 3.5.1; R Core Team, 2018).

To test the relationship between the Φ_PSII_ and the environmental conditions at the place of origin of the accessions, climate data made available by the Bergelson Lab (http://bergelson.uchicago.edu/) was used (Hancock et al., 2011). A linear regression model was applied for 293 of the accessions used in the GWAS. The model, performed with the basic R package, was calculated to explain the Φ_PSII_ of at each time point by the following variables as additive factors: annual mean temperature (“bio1_meant”), mean monthly temperature range (“bio2_diur_rng”), maximal annual temperature range (“bio7_temp_annrange”), mean temperature of the warmest quarter (“bio10_meanT_warm”), mean temperature of the coldest quarter (“bio11_meanT_cold”), latitude, longitude, number of consecutive days below 4°C (“FROSTY_DAY”), number of consecutive days above 0°C (“FROST_FREE”). For all of those factors a Kendall correlation with the Φ_PSII_ was performed per experimental time point.

The gene enrichment analysis was done with the Database for Annotation, Visualization and Integrated Discovery (DAVID) 6.8 (https://david.ncifcrf.gov/home.jsp). An EASE Score threshold, an adopted Fisher Exact test to estimate the likelihood of the annotation term, of 0.05 was chosen (Huang, Sherman, & Lempicki, 2009a, 2009b).

SNP‐based haplotype groups among 162 accessions for the *PSB27* gene (At1g03600) were determined with a Perl script (Kooke et al., 2016). The phenotypic association of the polymorphisms in *PSB27* were tested by grouping all accessions according to their allelic information per SNP and testing differences per genotype group and time point. The average values per genotype and time point were used. The parental accessions, Si‐0 and Ct‐1, were selected amongst their respective haplotype groups because of their high, respectively low, phenotypic values, seed availability, lack of vernalization requirement and relatively similar flowering time.

## 3 RESULTS

**FIGURE 1:**
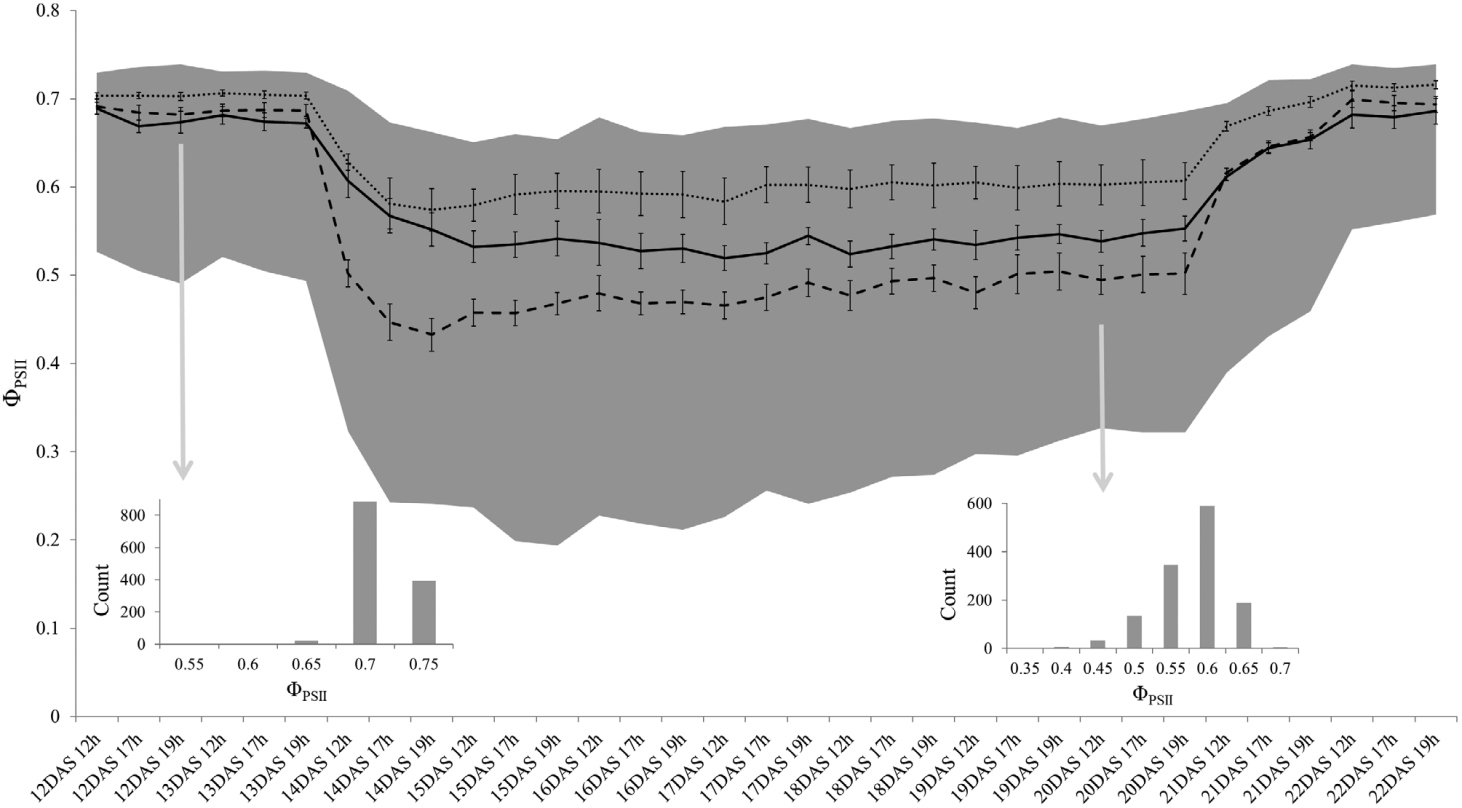
Response of Φ_PSII_ of the *A. thaliana* HapMap population to two changes in temperature between 21 and 5°C. The range of Φ_PSII_ within the population of 347 accessions is depicted in grey (defined by minimum and maximum individual values) for each time point over the course of 11 days measured at three time points per day. The average Φ_PSII_ values and standard deviation for Columbia‐0 (CS76113; full, black line) and two accessions with extreme phenotypes in the cold Catania‐1 (Ci‐1; dashed line) and Siegen‐0 (Si‐0; dotted line) are highlighted. All plants were grown in control conditions (21°C) until 13 days after sowing (13 DAS), on 14 DAS the temperature was lowered to 5°C upon light onset and kept constant until 20 DAS. On 21 DAS at light onset, the temperature was raised again to 21°C. For the 12 hr‐time point on the last day in control conditions (13 DAS) and the last cold day (20 DAS) the frequency distribution of Φ_PSII_ in the HapMap population is displayed

The HapMap population in *A. thaliana* is a frequently used, genetically optimized population with a large genetic and phenotypic diversity (Atwell et al., 2010; van Rooijen et al., 2017), however the response of this population to low temperature has not been studied yet. The response to a temperature decrease and subsequent increase was monitored in this population by CF imaging. The plants were first raised for 2 weeks at regular temperatures for *A. thaliana* growth (21°C air temperature, day and night) by which time most plants had formed their second true leaf pair (control treatment). To simulate an extreme, sudden, cold spell, the air temperature was reduced to 5°C (day and night; cold treatment). Four hours after the temperature decrease, the quantum yield of photosystem II (Φ_PSII_) of all genotypes was lower than in the control temperature (Figure 1). After 1 day at 5°C (15 days after sowing at 12 hr, 15DAS12h) more than 95% of all genotypes had a significantly lower Φ_PSII_ than on the same time of the day before the temperature reduction (13DAS12h); those genotypes that did not show a statistically significant reduction in Φ_PSII_ still had at least a 15% lower Φ_PSII_ on average in the cold. So there is an overall trend of all lines for an immediately lower Φ_PSII_ in the cold. Comparing all measurements of Φ_PSII_ made on that day (15DAS) compared to the last day of the control treatment (13DAS), the average Φ_PSII_ of the accessions was decreased by 23.1% (Table 1), with differences in Φ_PSII_ reduction ranging from 11.6 to 46.6% depending on the genotype. The range of Φ_PSII_ over the whole population also changed; in control conditions it ranged from 0.494 to 0.732 (13DAS) while after 1 day of low temperature the Φ_PSII_ ranged from 0.194 to 0.66. After 1 week of cold, the air temperature was raised to 21°C again (recovery treatment). On the 12 hr‐measurement of the day after the temperature increase (22 DAS), the Φ_PSII_ values had recovered to their pre‐cold values for 97.7% the genotypes. Although the Φ_PSII_ of the remaining genotypes did not recover fully, their Φ_PSII_ values still had increased by 14–38% compared to the last day of the cold treatment.

**TABLE 1:** Overview of the Φ_PSII_ values for the HapMap population

	Average Φ_PSII_	Min Φ_PSII_	Max Φ_PSII_	Reduction of Φ_PSII_ compared to control (%)	Φ_PSII_ H^2^
Last day control (21°C)	0.686 ± 0.024	0.494	0.732	—	0.18–0.22
Second cold day (5°C)	0.528 ± 0.07	0.194	0.66	23.1%	0.21–0.25
Seventh cold day (5°C)	0.56 ± 0.057	0.322	0.686	18.3%	0.15–0.18
Second day recovery (21°C)	0.695 ± 0.022	0.552	0.739	−1.3%	0.15–0.19

*Note:* The Φ_PSII_ values for the HapMap population were determined over 2 days of control temperature (21°C), 7 days of cold (5°C) treatment and two following days of recovery at 21°C. The average and standard deviation over three measurements per day in the respective condition, lowest and highest values within the population, the average Φ_PSII_ reduction in the cold compared to the control (21°C) in percent, and the ranges of broad sense heritability (H^2^) are given.

To determine if there is a relationship between the Φ_PSII_ determined in this experimental setup and the environmental conditions in the original habitat of the accessions a linear regression was modelled. The position (in longitude and latitude) of the sampling of the accessions in the wild and the respective annual temperatures and temperature range at this position were used to explain the Φ_PSII_ at the different experimental time points. The linear regression models were significant (*p*‐value <.001) for the time points in the cold (14 DAS to 20 DAS) with a R^2^ of approximately 0.17 and an adjusted R^2^ of approximately 0.15. The adjusted R^2^ values were lower in the control (*p*‐value >.05) and the recovery treatment, with approximately 0.02 and 0.03, respectively. Although for example, geographic position and the habitat‐temperature range can be predictors of Φ_PSII_ (depending on the time point), the strongest predictive factor is the average temperature in the coldest quarter. This factor is a significant negative predictor of the Φ_PSII_ value, however only under cold temperature (*p* value below .01 or below .001); it is not predictive of Φ_PSII_ in the 21°C control temperature period (12 and 13 DAS) and also not on the second day of recovery (22 DAS). Correlation analysis of Φ_PSII_ with the individual habitat‐factors tested in the linear model showed that at all time points in the cold there are significant negative correlations with the annual mean temperature (tau ~−0.13), the average temperature in the coldest quarter (tau ~−0.14) and the consecutive number of frost free days (tau ~−0.12). Equally, at all time points in the cold there was a positive correlation between Φ_PSII_ and the consecutive number of days below 4°C in the accessions habitats (tau ~0.15).

**FIGURE 2:**
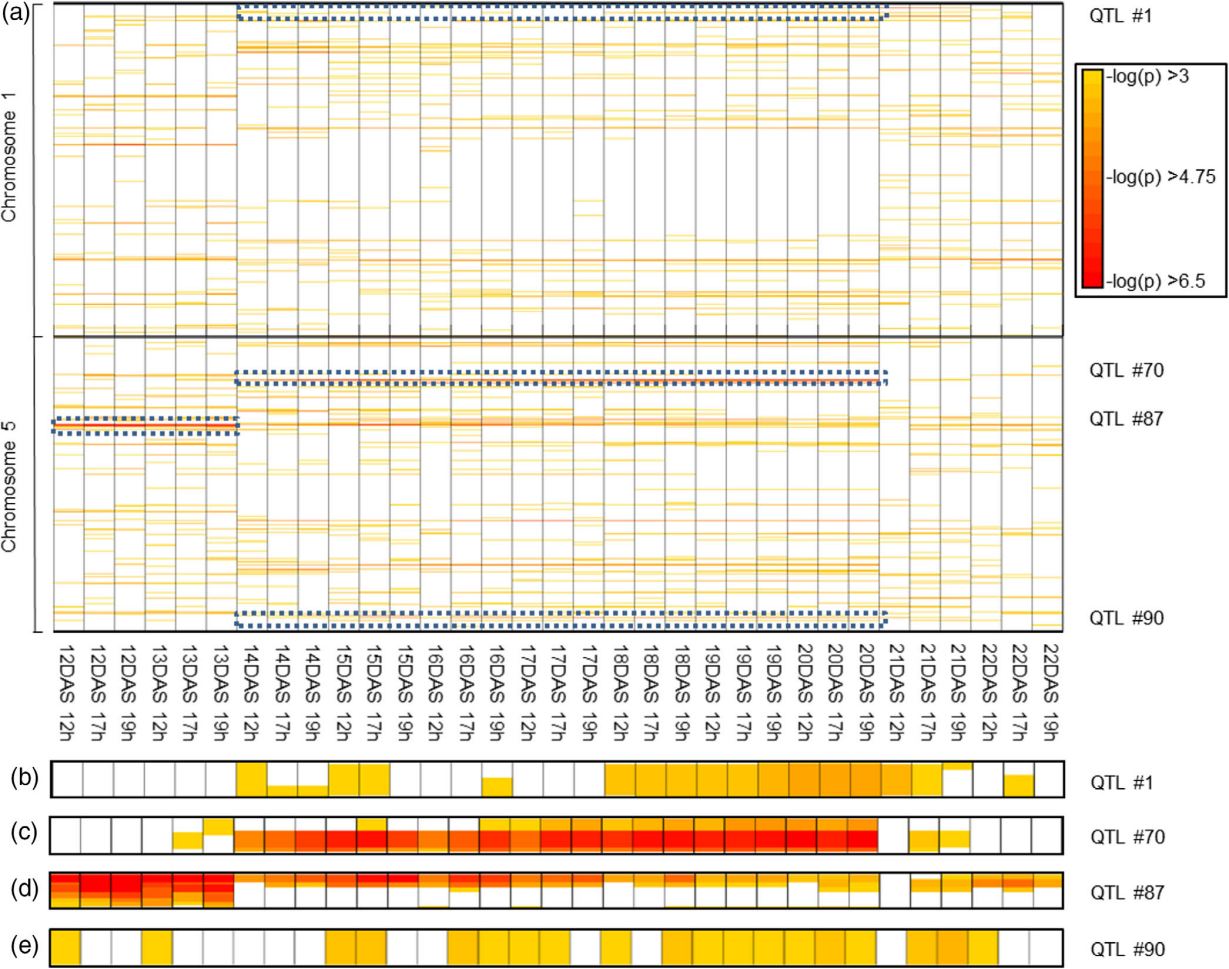
QTLs detected by association mapping on chromosome 1 and 5 in the *Arabidopsis thaliana* HapMap population. The heat map depicts the GWAS results of the association strength (−log_10_(p)) of the average Φ_PSII_ with the SNP information of the HapMap population (a). Each association of a –log_10_(p) > 3 at any given time point is indicated as a box at the physical genetic position (vertical axis) around the SNP. The GWAS analysis was performed for several time points at 12, 16, and 19 hr between 12 DAS and 22 DAS. Over the course of the experiment the temperature was changed: from 21°C (from 12 to 13 DAS) to 5°C (14 to 20 DAS), and back to 21°C (recovery; from 21 to 22 DAS). QTLs were defined as loci that were present on at least three time points in the control temperature conditions and 11 time points in the cold conditions. Of those, the QTLs #1, #70 and #90 in the cold condition and QTL #87 of the control condition are highlighted and shown in more detail (b–e). GWAS, Genome Wide Association Studies; QTL, quantitative trait locus

The broad sense heritability of the Φ_PSII_ value varied depending on the time points; in the control treatment (at 21°C) it ranged between 0.18 and 0.22 and from 0.15 to 0.27 in the cold. These are sufficiently high heritability values to allow a GWAS approach (van Rooijen et al., 2017). An association analysis was performed per time point. An overview of all loci at log10(p) ≥ 3 is shown in Figure S1. Further focus will be on chromosomes 1 and 5, containing two loci with a log_10_(p)‐value slightly above the very stringent Bonferroni threshold of 6.6; one at the control temperature (QTL # 87), the other at the low temperature (QTL # 70; Figure 2). The latter one exceeds the Bonferroni threshold at one single time‐point (DAS20 19 hr). Because of the low number of highly significant associations, priority was given to those associations that were reoccurring over time rather than appearing occasionally at higher significance. We therefore selected SNPs with a *p*‐value below 0.001 for at least half of the time‐points within a given treatment period (i.e. at least three time‐points for each of the 21°C‐treatments or 11 for the cold treatment). In the control treatment 105 SNPs were identified that meet these criteria; in the cold treatment 90 SNPs and in the recovery phase 94 SNPs were detected (Figure S1 and Table S1). Candidate genes were listed in a 3‐ kb region flanking each significantly associated SNP on either side. These lists of genes were compared across the three different conditions (control, cold, recovery) to determine if those candidate genes were common for the whole experiment or specific to one or two of the treatments. When comparing the genes in the control and cold, 1,415 genes were specific to the control condition and 1,309 genes were specific to the cold treatment. There were 638 genes specific to the recovery phase that were not present in any of the other two conditions. There are 207 genes common to all conditions, 711 that are common to the 21°C condition before (control) and after the cold treatment (recovery); and 374 and 341 genes that are common to the control and the cold treatment or to the cold and the recovery treatment, respectively. We focussed on the candidate genes specific to the cold treatment. A gene enrichment analysis shows that there is an overrepresentation of photosynthesis or chloroplast related genes. In the category 'cellular location' 2.5–16.4‐fold enrichment was found for chloroplast photosystem II, chloroplast ribulose bisphosphate carboxylase complex and chloroplast thylakoid lumen (*p*‐values between .012 and .4; Figure S2).

**FIGURE 3:**
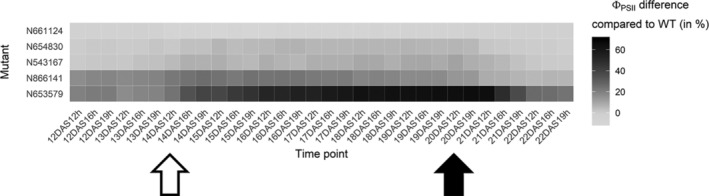
Φ_PSII_ difference of five *A. thaliana* mutants compared to the Col‐0 wild type. Mutants for the genes *PSB27* (At1g03600; mutants N653579 and N654830), a RING/U‐box superfamily gene (At5g12310), *ASN2* (At5g65010) and *DGS1* (At5g12290) were characterized amongst others in a low temperature experiment. The Φ_PSII_ differences of the respective T‐DNA mutants N653579, N654830, N661124, N543167 and N866141 are expressed as relative values compared to their genetic wild type (WT) background, Col‐0. These relative values are calculated per time point over the course of the experiment: in the control condition before the cold treatment (12 and 13 DAS), during the cold treatment (14 DAS to 20 DAS) and in the control condition after the cold treatment (21 and 22 DAS). The time points of temperature change are indicated by arrows, an open arrow indicates the onset of the cold treatment and a filled arrow the raise of temperature at the end of the cold period. Three Φ_PSII_ measurements were taken per day in the light at ~12, 16 and 19 hr. The Φ_PSII_ values of the mutant N653579 and N866141 differ from those of Col‐0 at all time points (*p* < 0.001). The mutant N661124 is different from Col‐0 at 14 of 21 time points in the cold (*p* < 0.05) and N543167 at all time points in the cold (*p* < 0.05)

In order to validate any of the candidate genes in the regions covered by the QTLs, which may be responsible for the genetic variation displayed in Φ_PSII_ at low temperatures, 31 T‐DNA insertion lines for 24 candidate genes were analysed. From the list of genes in the region covered by QTLs identified for Φ_PSII_ in the cold, those 1,309 that are unique to the cold treatment were to us of main interest. Further refinement of the candidate list was mainly done via the functional annotation in The Arabidopsis Information Resource database (TAIR, www.arabidosis.org) for genes that were involved in stress response, in signal transduction or in photosynthesis and associated metabolic functions (Table S2), and mutants were screened for phenotypic differences for Φ_PSII_ upon cold exposure_,_ when compared to wild‐type plants. Two prominent candidate genes for the highly significant cold‐specific QTL #70, are At5g12310 and At5g12290. While the SNP with the highest association identifying this QTL is in At5g12310, encoding a predicted RING/U‐box superfamily protein, the mutant, N661124, showed only a marginal increase in Φ_PSII_ compared to that of the wild type, on average 7% in the cold (*p* < .05 for 14 out of 21 time points in the cold; Figure 3). Instead, mutant N866141, corresponding to *DGS1 (DIGALACTOLIPID‐DEFICIENT MUTANT 1 SUPPRESSOR 1*; At5g12290), had on average a 20% lower Φ_PSII_ than the wild type in the cold (significant difference of *p* < 7 × 10^−5^ at all time points in the cold). The mutant should be compared ideally to Col‐3 and not Col‐0, however, since Col‐3 and Col‐0 are phenotypically similar, we consider the difference in Φ_PSII_ sufficiently large to assume the *DGS1* gene is involved in the cold response. The *ASPARAGINE SYNTHETASE 2* gene (*ASN2*, At5g65010), was a candidate for the cold‐treatment QTL #90. A mutant for this gene, N543167, had on average an 8% lower Φ_PSII_ in the cold treatment (at least *p* < 0.04 and for 13 time points even higher, *p* < 0.001). Finally, T‐DNA insertion mutant N653579 for the *PHOTOSYSTEM* “*B*” (*II*) *GENE 27* (*PSB27*, At1g03600, cold QTL #1), showed the strongest reduction in photosynthesis efficiency of all tested mutants; in control conditions the Φ_PSII_ of N653579 was on average 20% lower than that of Col‐0 while in the cold it was on average 53% lower (*p* < 2 × 10^−30^). T‐DNA insertion line N654830, containing another T‐DNA insertion in the same gene, showed only a slight reduction in Φ_PSII_, in the cold, compared to the wild type. However, since this insertion is in the presumed promotor region of the *PSB27* gene, its function may be hardly affected by the mutation. N653579 carries a knock‐out allele of the *PSB27* gene that was described to have a role in light adaptation (Hou, Fu, Garcia, Buchanan, & Luan, 2015). *PSB27* is also one of four genes found in the enriched cellular localisation categories “Chloroplast photosystem II/ thylakoid (/lumen).”

**FIGURE 4:**
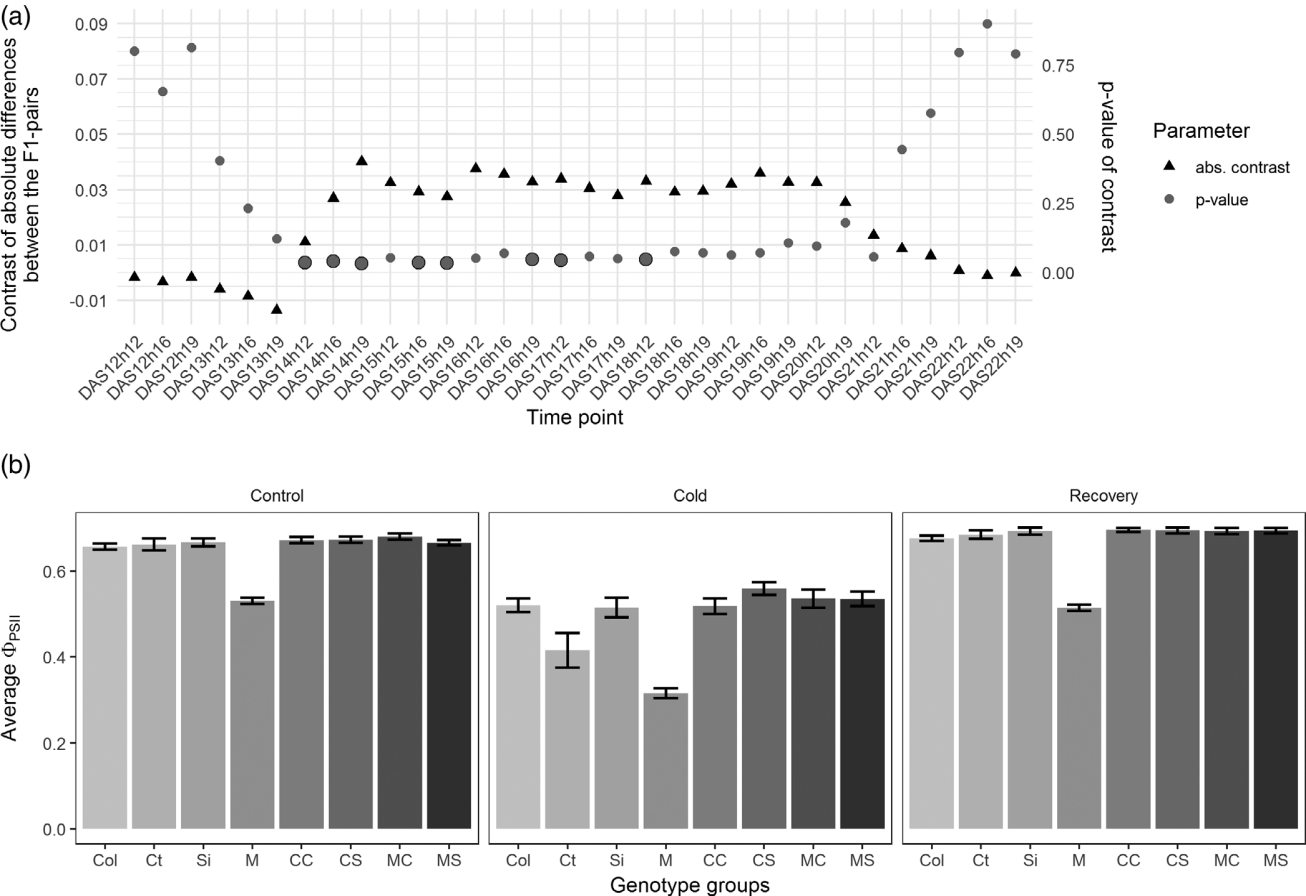
Complementation test for the mutant N653579. Two accessions with different *PSB27* (At1g03600) alleles, Siegen (“Si”; Si‐0) and Catania (“Ct”; Ct‐1) were crossed with the knock‐out mutant N653579 (“M”). The genetic background of the mutant is the wild‐type accession Columbia (“Col”; Col‐0) which was also crossed with the two accessions Si‐0 and Ct‐1. To test the significance in difference of the average Φ_PSII_ between the F1 plants from the two pairs of crosses N653579 × Si (“MS”) versus N653579 × Ct (“MC”) and Col × Si (“CS”) versus Col × Ct (“CC”) the significance of the contrast, (mean(CC) – mean(CS)) – (mean(MC) − mean(MS)), was determined per time point. The absolute difference in mean Φ_PSII_ between those groups (|(CC‐CS)| − |(MC‐MS)|) ranges between −0.0136 and 0.0402 per time point (“abs. Contrast” depicted as triangles; a). The significance of the contrast is shown by dots, in case of a *p*‐value below 0.05, the dots are larger with a border line. The average Φ_PSII_ per genotype (and 2*SE) is displayed for three specific time points 13DAS19h (Control), 14DAS19h (Cold) and 22DAS19h (Recovery; b). The Φ_PSII_ of all lines decreases in low temperature and there is a tendency for a higher ΦPSII in CS compared to other F1 plants in the cold which is apparent on time point 14DAS19h

We further examined the genetic variation in *PSB27*, to validate that it indeed explains the variation in Φ_PSII_ associated with QTL #1. At gene At1g03600, an allelic difference at one non‐synonymous SNP at position 899,112 (T or C, which leads to an amino acid change from alanine to a valine) is associated with a difference in phenotype at all of the time points in the cold (*p*‐value ranging from 0.0184 to 0.0004 on the different time points). The accessions with a T‐allele (79 out of 162 accessions) had an average Φ_PSII_ value of 0.686 ± 0.001 under standard temperature (21°C) and 0.55 ± 0.001 in the cold, while accessions with a C‐allele (83 accessions) had an average Φ_PSII_ of 0.682 ± 0.001 and 0.534 ± 0.001, respectively. Finally, a quantitative complementation test (Mackay, 2001; Weigel, 2012) was performed to confirm that allelic changes at the *PSB27* gene cause the phenotypic variation. Two accessions were selected to serve as parents in crosses to complement the mutant phenotype, Siegen‐0 (Si‐0) and Catania‐1 (Ct‐1), with Si‐0 representing the phenotypic class with the higher Φ_PSII_ (T at 899112) in the cold treatment and Ct‐1 the group with a lower Φ_PSII_ (C at 899112; Figure 1). For the complementation test, the different alleles under investigation are assessed in the heterozygote state to normalize the effect of genetic variation residing at other loci (Weigel, 2012). Thus, the accessions Si‐0 and Ct‐1 were each crossed with the homozygous T‐DNA insertion knock‐out *psb27*‐mutant N653579 as well as with the Col‐0 wild type. If the difference in Φ_PSII_ of the F1s between the selected accessions and Col‐0 is significantly different from the difference in Φ_PSII_ of the F1s between the same accessions and the KO mutant this difference can only be caused by the allelic variation of the gene under investigation. A difference between the absolute mean differences of the pairs shows that in the cold the difference between the F1 pairs in the Col‐0 background is larger than in the mutant background (Figure 4). In the cold, the absolute difference in Φ_PSII_ between Col × Si (“CS”) and Col × Ct (“CC”) is between 0.01 and 0.04 larger than between N653579 × Si (“MS”) and N653579 × Ct (“MC”). CS has on average a higher Φ_PSII_ than CC. However, the significance for this difference in contrast between the two pairs varies at a *p*‐value between 0.03 and 0.9 (with a *p*‐value below 0.05 on 8 out of 20 time points in the cold treatment). Therefore, only a tendency can be reported. This tendency supports, however, the proposition that natural variation at the *PSB27* gene (At1g03600) has an influence on the Φ_PSII_ cold response and that it can be responsible for the observed variation attributed to QTL#1.

## 4 DISCUSSION

In this study the effect of cold on natural accessions of *Arabidopsis thaliana* was examined by the means of CF imaging of photosynthesis efficiency. To ensure highly stable environmental conditions, the plants were grown in a closed growth room with controlled air temperature, light conditions, relative humidity and a hydroponic system that allowed the control of water and nutrient supply. These measures all contributed to reducing the variability and potentially disturbing effects of the environment on the genetics of the environmentally sensitive trait Φ_PSII_. All genotypes were monitored over the complete duration of the experiment with three measurements made per day. These repeated measurements enabled us to identify persistent patterns over the course of time that are specific to the respective conditions, reducing the effect of errors that could come from individual observations.

The daily measurements allowed us also to visualize the progressive response of the plants to low temperature. Photosynthesis efficiency responded immediately to the lowering of the temperature in all genotypes. The severity of the impact of the temperature change on the absolute value of Φ_PSII_ depended on the genotype. After 1 day of low temperature exposure, the overall photosynthetic efficiency did not decrease further during 1 week of cold treatment. So the effect on light‐use efficiency seems to be immediate and does not show any long‐term effects in the ‐ day cold exposure period. This is in agreement with the measurements of Strand, Hurry, Gustafsson, and Gardeström (1997) on leaves of Col‐0 plants that were exposed to low temperatures. After three and 10 days at 5°C, the maximum quantum yield of photosystem II, *F*_*v*_/*F*_*m*_, stayed nearly the same (ca. one third of the value at the control growth temperature), and no further reduction was seen within the 7 days of cold. In our experiment, we increased the temperature after 1 week of cold exposure and observed a nearly full recovery of photosynthesis efficiency after 1 day. This implies that the plants were not permanently damaged by the low temperature. Such a recovery is consistent with the ecology of *A. thaliana*, which grows in temperate regions where exposure to low temperatures in the growing cycle is common. The species is known to have mechanisms to adapt to cold and even acclimate to freezing temperatures (Alonso‐Blanco et al., 2005; Oakley et al., 2018; Schulz, Tohge, Zuther, Fernie, & Hincha, 2016). *A. thaliana* is shown to be chilling tolerant and after prolonged exposure to low temperatures a recovery of photosynthesis has been observed that is due to a change in the regulation of photosynthesis (Holaday, Mahan, & Payton, 2016; Strand et al., 1997). The linear regression model and the correlation analysis relating the Φ_PSII_ determined for accession in this experiment with the habitat environmental factors point to a general trend that accessions with low Φ_PSII_ under the cold conditions in this experiment come from habitats with shorter and milder winter periods. While much more work would be needed to confirm a selective advantage of certain genotypes in certain habitats, this observation suggests there may well be an adaptive mechanism in place that provides some accessions from colder climates with more efficient photosynthesis at mild (sub‐zero) low temperature exposure than other accessions.

The photosynthetic efficiency measure we used, Φ_PSII_, reflects not only on a local photosynthetic process but also the broader physiological state of the plants and responds to several environmental changes, including cold (Baker & Rosenqvist, 2004; Roháček, Soukupová, & Barták, 2008). It has already been used for genetic analysis (Fracheboud, Ribaut, Vargas, Messmer, & Stamp, 2002; Prinzenberg, Víquez‐Zamora, Harbinson, Lindhout, & van Heusden, 2018; van Rooijen et al., 2017), but no genome wide association study was done for this trait to investigate low temperature responses. The Φ_PSII_ data of the HapMap population was used for a genetic analysis per time point and several associated loci were identified, both those common to all treatments and those that are specific to a treatment. Amongst the candidate genes, photosynthesis‐related factors are found to be enriched for the response to cold, which is consistent with our quantification of the photosynthetic trait, Φ_PSII_. In addition, Ф_PSII_ is very sensitive to low temperature exposure. It was hence not surprising, that candidate genes that are already described in photosynthetic adaptation are also candidate for the cold response of Ф_PSII_. Protection of the photosynthesis apparatus, the primary energy acquiring process of the plant, against diverse and changeable environmental conditions is essential for plant survival. In lower temperatures, the overall metabolic activity of the plant is reduced. If photosynthesis efficiency is not downregulated, high excitation pressure and sink limitations in low temperature can lead to the enhanced production of reactive oxygen species (Juszczak, Cvetkovic, Zuther, Hincha, & Baier, 2016; Triantaphylidès et al., 2008) and photodamage (Adam & Murthy, 2014; Krieger‐Liszkay, 2005; Suzuki & Mittler, 2006; Wise, 1995). Several processes could play a role in photoprotection in the cold, like energy dissipation from the photosystem by non‐ photochemicalquenching (Kościelniak & Biesaga‐Kościelniak, 2006; Müller, Li, & Niyogi, 2001; Oquist, Hurry, & Huner, 1993), changes in pigment content (Fracheboud et al., 2002; Huner, Elfman, Krol, & McIntosh, 1984; Schöner & Heinrich Krause, 1990), accumulation of osmolytes (DeRidder & Crafts‐Brandner, 2008; Holmström, Somersalo, Mandal, Palva, & Welin, 2000) or changes in membrane fluidity and its associated proteins (Takami, Shibata, Kobayashi, & Shikanai, 2010). A fine‐tuned regulation of photosynthesis and photoprotective mechanisms is necessary to prevent permanent damage to the photosystem, while still enabling sufficient photosynthetic operation for the production of photoassimilates. To allow this fine‐tuning a multifactorial cold response of photosynthesis would be a likely strategy and a polygenic control of the photosynthetic response to cold is expected. Therefore, and not unexpectedly, many QTLs were identified, most with low association‐strength. Only two loci exceeded the very conservative Bonferroni threshold. It illustrates the highly polygenic nature of the trait, which together with the modest broad sense heritability, strongly reduces the statistical power in GWAS to detect significant QTLs. The use of a stringent cut‐off minimizes the detection of false positives but will also exclude many small‐effect QTLs that are thought to be one of the main sources of missing heritability in quantitative genetic studies (Bloom, Ehrenreich, Loo, Lite, & Kruglyak, 2013). To overcome this, we gave more weight to the persistence of the association over time, rather than to the strength of the association, in the selection of candidate genes. No major, common genetic factor involved in the low temperature regulation of photosynthesis stands out in our study of the worldwide collection of genetic variants in *A. thaliana*. Instead there appears to be a much more multifactorial regulation, possibly reflecting the presence of different cold adaptation mechanisms, each with comparable representation in the germplasm. This is supported by the transcription analysis of 10 *A. thaliana* accessions which showed large natural variation in response to low temperature, with 75% of the differentially expressed genes to be genotype specific (Barah et al., 2013).

Genetic confirmation of multifactorial, allelic variation is not trivial, especially of small‐effect loci. Studying knock‐out mutants is one possible way to confirm the involvement of a candidate gene that was discovered by statistical association. However, natural allelic variation detected in a QTL study cannot always be proven by a mutant analysis, as for example, gene redundancy or genotype‐specific pleiotropic effects may mask the mutant phenotype. Therefore, we decided to support the candidate gene study by another methodology. Complementation tests or the cloning and transformation of one allelic variant into a different genetic background is a way to look at functional differences compared to a loss‐of‐function effect. However, in these cases pleiotropic effects, heterosis or the genomic position of the transgene can also influence the phenotype and the outcome of the analysis. Nevertheless, due to the large number of available mutants for *A. thaliana*, albeit mostly in the Col‐0 background, a large number of candidate genes can be screened relatively easily. In combination with a complementation test, this can provide sufficient proof to determine if an allelic variant is causal to the QTL effect. Our mutant study indicated the involvement of several genes in the photosynthesis phenotype. Two mutants that showed a significant and sizeable difference in Φ_PSII_ compared to the wild type are *dgs1* and *asn2*, that are candidates for the QTLs #70 and #90 respectively. Both genes, *DGS1* and *ASN2*, were previously also identified as candidates underlying QTLs involved in the adaptation of Φ_PSII_ to high‐light stress conditions (van Rooijen et al., 2017). *DGS1* is important for lipid remodelling, in this case the conversion of phospholipids to galactolipids, which is important for high‐light stress response (van Rooijen, Harbinson, & Aarts, 2018). *ASN2* is involved in nitrogen allocation and the mutant was shown to have a lower leaf nitrogen concentration and a pale, low‐chlorophyll phenotype; however no effect on photosynthetic CO_2_ assimilation was found under non‐stressed growing conditions (Gaufichon et al., 2013). Both genes are strong candidates to be involved in the photosynthetic response to cold stress. Exposure to low temperatures can cause over‐excitation of the photosystem, like high‐light stress, and similar protective mechanisms can apply.

One candidate gene was confirmed to be underlying one of the QTLs. The Φ_PSII_ of the *psb27* mutant, corresponding to cold‐QTL #1 on chromosome 1, was much lower than that of the wild type. The subsequent quantitative complementation test supports the influence of variation at *PSB27* to affect Φ_PSII_ in the cold. Among the HapMap population, haplotypes with a thymine instead of a cytosine at SNP 899,112, which is in the *PSB27* ORF, had a slightly (between 2 and 5%), but significantly (*p*‐value < 0.05) higher Φ_PSII_. In the complementation test, accessions Si‐0 and Ct‐1, carrying the contrasting alleles, were crossed with the *psb27*‐mutant. The F1s have a significantly higher Φ_PSII_ than the mutant. This indicates that both alleles are functional, which is expected of natural alleles of genes for which a knock‐out mutant gives a strong fitness‐decreasing phenotype. Based on the comparisons of the F1s between accessions, with the Col‐0 × Si‐0 F1 resembling Si‐0 and Col‐0 × Ct‐1 F1 resembling more Col‐0 than Ct‐1, it could be that the dominance of the different tested *PSB27*‐alleles is the following: the Si‐0 alleles (*PSB27^Si‐0^*) could be dominant over the other alleles with the mutant in the Col‐0 background (*psb27* ^Col‐0^) being the least dominant (*PSB27* ^*Si‐0*^ > *PSB27* ^*Col‐0*^ > *PSB27* ^*Ct‐1*^ > *psb27* ^*Col‐0*^ *)*. In the wild‐type background, the F1 from the cross Col‐0 × Si‐0 had a tendency for a higher Φ_PSII_ than the F1 from the cross to Ct‐1. This is in accordance with the phenotypic difference observed between Si‐0 and Ct‐1. There is no difference between the Si‐0‐ and Ct‐1‐ F1 plants in the mutant (N653579) background. As the effect in the heterozygote state shows a different tendency in a combination with a wild‐type Col‐0 than with a mutant (psb27‐Col‐0) background, the complementation test supports the conclusions from the association mapping that allelic variation of the *PBS27* gene impacts Φ_PSII_ in the cold. The allelic effect of this locus on Φ_PSII_ may be too small to be consistently validated over all time points in the heterozygous background. Heterozygosity effects due to the mixed genetic backgrounds of the F1 plants may mask the gene effect. Furthermore, additional mutations or differences between the Col‐0 used for the mutant and for the F1crosses could also increase the phenotypic variability. Another mutation is known to exist in N653579 (in the gene *CP26*), however in earlier studies this was shown not to influence Φ_PSII_ (Hou et al., 2015). CP26 is also not among the candidate genes identified in the GWAS analysis. Furthermore, we found another T‐DNA insertion allele of *PSB27* to also show a lower Φ_PSII_ in the cold, though with a much less drastic difference than seen in N653579. Taking together the results from the mutant analysis and the complementation test, *PSB27* seems to influence photosynthetic responses to cold. *PSB27* codes for a thylakoid lumen protein, associated with the PSII complex. In cyanobacteria, the PSB27 protein is involved in the repair and biogenesis of the PSII protein complex, transiently binding the photosystem II complex subunits D1 and CP47 (Cormann, Möller, & Nowaczyk, 2016; Liu, Huang, Chen, Gross, & Pakrasi, 2011; Liu, Roose, Cameron, & Pakrasi, 2011) and facilitating the assembly of the water oxidizing complex (Komenda et al., 2012; Roose & Pakrasi, 2008). Hou et al. (2015) studied the mutant N653579 and showed that the loss of the PSB27 protein caused an overall lower Φ_PSII_, growth retardation and pigment aberration in plants grown in fluctuating light when compared to the wild type. Though the exact function of PSB27 in PSII repair upon stress response is not confirmed in *A. thaliana*, there is a known involvement in fluctuating light adaptation. The temperature and high light stresses are connected. PSII can easily be damaged at low temperatures by high excitation pressure caused by light (Distelbarth, Nägele, & Heyer, 2013). Correspondingly, the photo‐repair function of PSB27 may also contribute to the adaptation to low temperatures.

This study showed that the measurement of Φ_PSII_ in a diversity panel of *A. thaliana* suitable for GWAS is very effective in the identification of many genetic loci involved in photosynthesis response to cold. Environmental stability and especially the repeated measurements over time sufficiently repressed the non‐genetic environmental effect on the phenotypic variation to allow the detection of these low‐effect QTLs. To further study this genetic variation, and identify causal genes, a reduction in genetic complexity will be useful, for example, by examining biparental (Brachi et al., 2010), multiparent (such as MAGIC; Kover et al., 2009; Zheng, P Boer, & van Eeuwijk, 2014) or regional populations (Long et al., 2013). We assume that the genetic regulation in the *A. thaliana* HapMap population is complex and guided by different, multigenic pathways. Nevertheless, at least one candidate gene could be validated. This interdisciplinary approach, combining photosynthesis research and genetics, revealed a new molecular component, PSB27, to be of relevance in cold stress response.

## CONFLICT OF INTERESTS

The authors declare no conflicts of interest.

## AUTHOR CONTRIBUTIONS

Aina E. Prinzenberg planned and supervised the experiments, performed the GWAS experiment, part of the mutant analysis and complementation test. Lucia Campos‐Dominguez contributed to the mutant analysis and the complementation test. Willem Kruijer performed the GWAS analysis, made the corresponding heatmap and provided advice on the statistical analysis. Jeremy Harbinson and Mark G. M. Aarts initiated the project, assisted in project planning and advised on photosynthetic and genetic issues, respectively. Jeremy Harbinson did the enlargements for the Figure 2. All authors contributed to the writing of the manuscript.

## Supporting information


**FIGURE S1.** QTL detected by association mapping with the *Arabidopsis thaliana* HapMap population. The heat map depicts the association strength (−log_10_[p]‐score) of the average Φ_PSII_ with the SNP information of the HapMap population. On the horizontal axis the genome of *Arabidopsis thaliana* (SNP locations) with the chromosomes 1 to 5 is shown from left to right is shown. On the vertical axis are the time points from 12 DAS to 22 DAS at 12, 16, and 19 hr. The data in control temperature is from 12 to 13 DAS, for the cold condition from 14 to 20 DAS and for the control treatment after cold (recovery) from 21 to 22 DAS. SNPs with a −log_10_(p)‐score above 3 are indicated by a coloured box from yellow as lowest association and red as highest (see legend). Numbered blue lines indicate the position of the QTL that were present on at least 3 time points in the control‐temperature treatments and on at least 11 time points in the cold treatment.

**FIGURE S2.** GO term enrichment of candidate genes. The treatment specific QTL identified in the GWAS study comprise 1,415 genes specific to the control condition, 1,309 specific for the cold and 638 genes specific only to the recovery condition. A gene enrichment analysis was performed for each of the condition specific gene sets with “David 6.8” for biological process, cellular localisation and molecular function. The *PSB27* gene (At1g03600) is represented in the cold specific category “cellular localisation” (chloroplast photosystem II, chloroplast thylakoid lumen, thylakoid).

**FIGURE S3.** Steady‐state quantum yield of photosystem II electron transport (Φ_PSII_) in the cold of different lines of Col‐0 wild type. Three lines of Col‐0 were monitored for their Φ_PSII_ over the whole temperature experiment: the plants were grown until 14 days after sowing (14 DAS) in 21°C and then exposed to an air temperature of 5°C for seven days. On day 21 after sowing (21 DAS) the temperature was raised again to 21°C. Three measurements of Φ_PSII_ were done per day at 12 h, 17 h and 19 h and the average and standard error of 6 to 8 replicates of each line per time point are shown. The lines N907 and N76113 were compared to N60000 with a Student's *t* test and were not different from N60000 (*p* > .15 and *p* > .36, respectively) on any of the time points between 12DAS and 22DAS.

**TABLE S1.** QTLs detected by association mapping in *Arabidopsis thaliana HapMap population*. To select QTL, associations with a –log(p) > 3 were selected that were present in 3 out of 6 time points in the 21°C treatments (control and recovery) and 11 out of 21 time points in the cold treatment. These regions were numbered consecutively according to their occurrence on the physical map (1 to 105, 90 and 94 respectively for the three conditions). A 60 kbp interval around the associated SNP was chosen for the selection of candidate genes, in case those intervals were overlapping they are listed as a single QTL (therefore QTLs can be larger than 60 kbp). For each condition, control, cold and recovery, and QTL the chromosome and start and end position in bp is given.

**TABLE S2.** The list of 31 analysed mutants for cold‐specific QTLs and their Φ_PSII_ compared to the wild type. All mutants that were assessed for their ΦPSII‐response to low temperature. They are listed with the gene and mutant name, the left border and right border primer that was used for verification of the mutation, the insert location according to the TAIR webpage (https://www.arabidopsis.org), gene function according to TAIR, the number of the cold specific QTL and the respective genetic background accession of the mutant. For each mutant and time point the difference in ΦPSII between the mutant and the wild type is given in percent and the p‐value for the difference. The mutant N654830 was not genotyped, it was ordered as homozygote mutant. The primers were designed with the help of the webtool OligoCalc (http://biotools.nubic.northwestern.edu/OligoCalc.html) or directly with the primer design tool from SALK (http://signal.salk.edu/tdnaprimers.2.html). The primers LBb1.3 or o2588 were used for the verification of the T‐DNA insert of the SALK‐ or Gabi‐Kat lines, respectively.

**TABLE S3.** List of *Arabidopsis thaliana* accessions analysed in the GWAS. List of all genotypes of the *A. thaliana* HapMap population that were evaluated in the GWA study.
