# Supplementary figures and images for: Natural variation of photosynthetic efficiency in Arabidopsis thaliana accessions under low temperature conditions
Source: Plant Cell Environ. 2020 Jun 28;43(8):2000–13. doi: 10.1111/pce.13811 (PMC7497054; doi:10.1111/pce.13811)

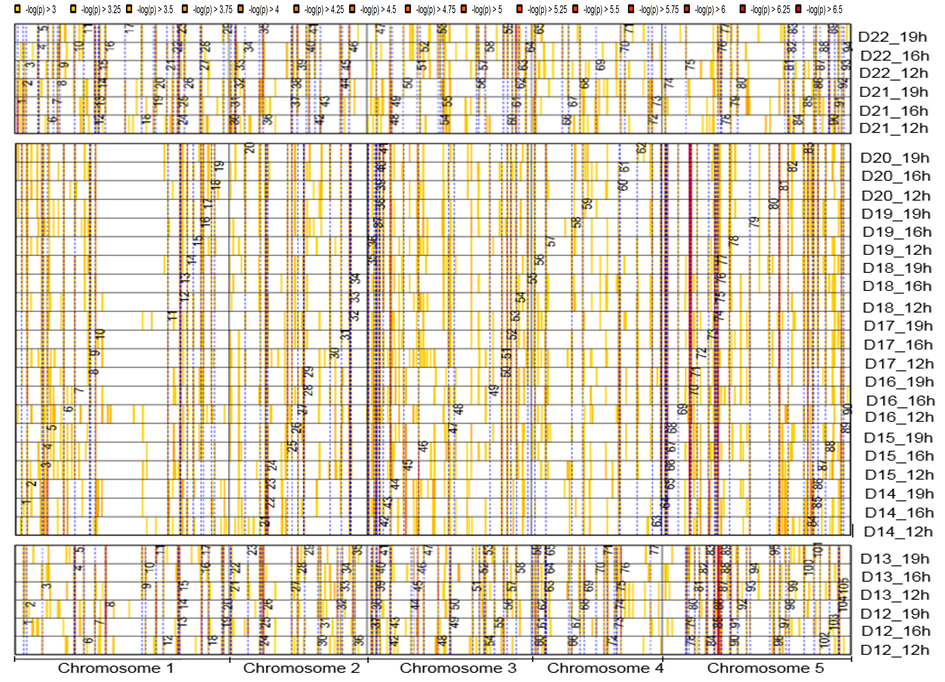

Supplement: Supplementary file 1 — FIGURE S1. QTL detected by association mapping with the Arabidopsis thaliana HapMap population. The heat map depicts the association strength (−log10[p]‐score) of the average ΦPSII with the SNP information of the HapMap population. On the horizontal axis the genome of Arabidopsis thaliana (SNP locations) with the chromosomes 1 to 5 is shown from left to right is shown. On the vertical axis are the time points from 12 DAS to 22 DAS at 12, 16, and 19 hr. The data in control temperature is from 12 to 13 DAS, for the cold condition from 14 to 20 DAS and for the control treatment after cold (recovery) from 21 to 22 DAS. SNPs with a −log10(p)‐score above 3 are indicated by a coloured box from yellow as lowest association and red as highest (see legend). Numbered blue lines indicate the position of the QTL that were present on at least 3 time points in the control‐temperature treatments and on at least 11 time points in the cold treatment. [file PCE-43-2000-s001.tiff]

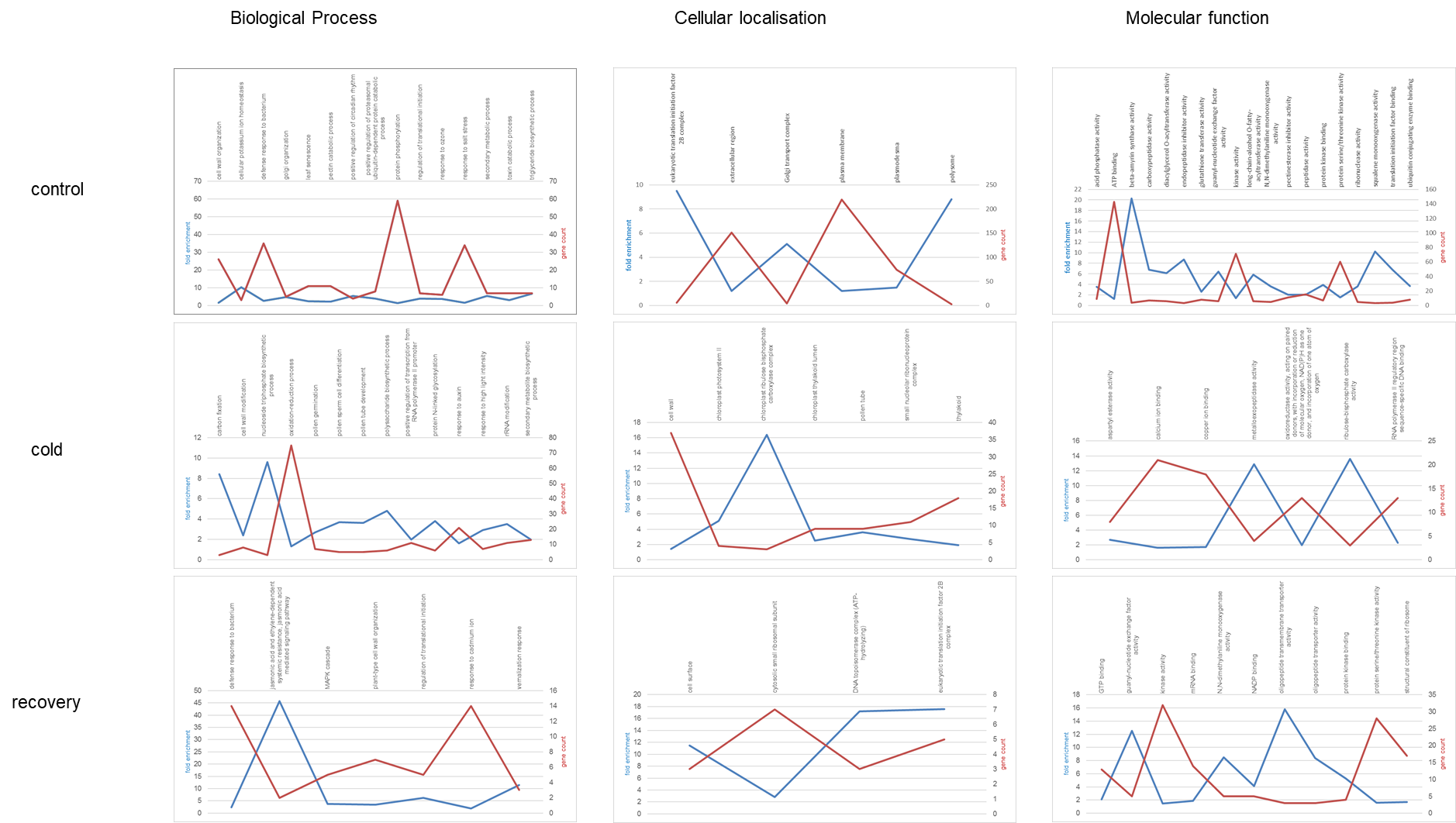

Supplement: Supplementary file 2 — FIGURE S2. GO term enrichment of candidate genes. The treatment specific QTL identified in the GWAS study comprise 1,415 genes specific to the control condition, 1,309 specific for the cold and 638 genes specific only to the recovery condition. A gene enrichment analysis was performed for each of the condition specific gene sets with “David 6.8” for biological process, cellular localisation and molecular function. The PSB27 gene (At1g03600) is represented in the cold specific category “cellular localisation” (chloroplast photosystem II, chloroplast thylakoid lumen, thylakoid). [file PCE-43-2000-s002.tiff]

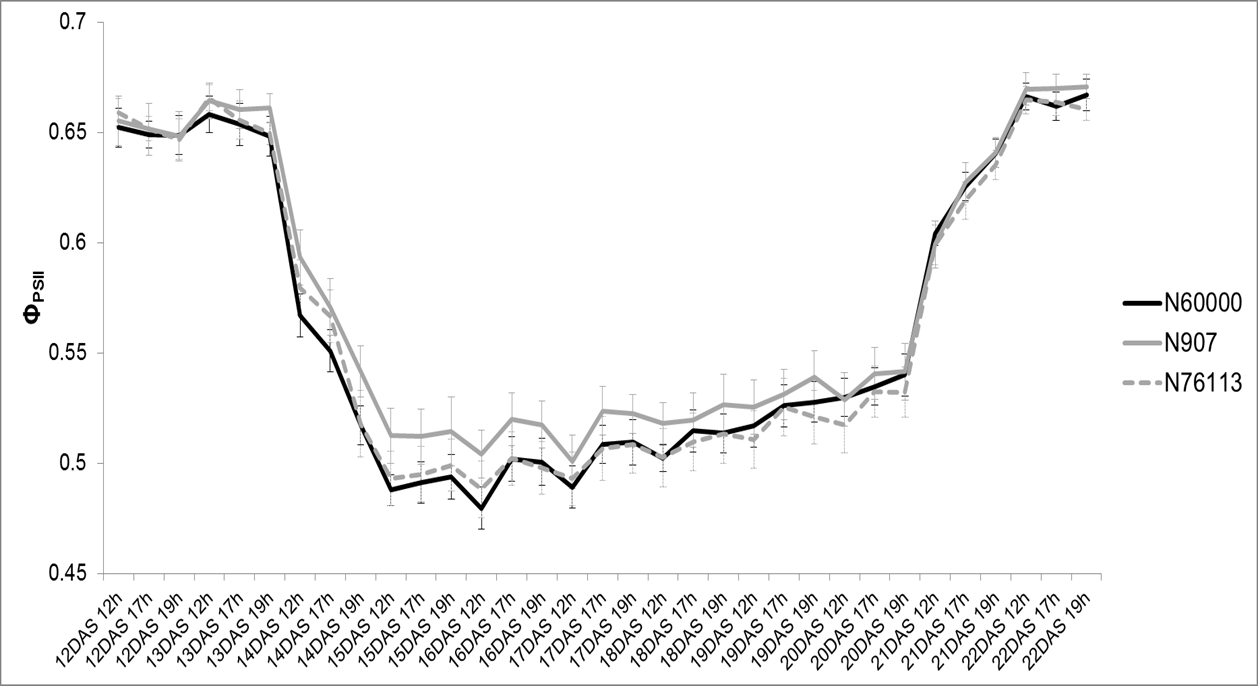

Supplement: Supplementary file 3 — FIGURE S3. Steady‐state quantum yield of photosystem II electron transport (ΦPSII) in the cold of different lines of Col‐0 wild type. Three lines of Col‐0 were monitored for their ΦPSII over the whole temperature experiment: the plants were grown until 14 days after sowing (14 DAS) in 21°C and then exposed to an air temperature of 5°C for seven days. On day 21 after sowing (21 DAS) the temperature was raised again to 21°C. Three measurements of ΦPSII were done per day at 12 h, 17 h and 19 h and the average and standard error of 6 to 8 replicates of each line per time point are shown. The lines N907 and N76113 were compared to N60000 with a Student's t test and were not different from N60000 (p > .15 and p > .36, respectively) on any of the time points between 12DAS and 22DAS. [file PCE-43-2000-s003.tiff]
